# High serum ferritin is associated with genetic instability in myelodysplastic neoplasms

**DOI:** 10.1007/s00432-026-06457-1

**Published:** 2026-04-09

**Authors:** Christina Ganster, Hannes Treiber, Gina Westhofen, Fabian Beier, Tienush Rassaf, Haifa Kathrin Al-Ali, Reingard Stuhlmann, Bertram Glass, Ulrike Bacher, Katayoon Shirneshan, Tim H. Brümmendorf, Ulrich Germing, Norbert Gattermann, Detlef Haase

**Affiliations:** 1 INDIGHO Laboratory, Department of Hematology and Medical Oncology, University Medical Center Göttingen (UMG), Robert-Koch-Str. 40, 37075 Göttingen, Germany; 2 Department of Hematology, Oncology, Hemostaseology, and Stem Cell Transplantation, RWTH Aachen University Hospital, Aachen, Germany; 3 Center for Integrated Oncology, Aachen Bonn Cologne Düsseldorf (CIO ABCD), Aachen, Germany; 4 Department of Cardiology and Vascular Medicine, Medical Faculty, West German Heart and Vascular Center, University Hospital Essen, Essen, Germany; 5 University Halle, Krukenberg Cancer Center Halle, Halle (Saale), Germany; 6 Onkologisch-Hämatologisches Zentrum Wendland, Dannenberg, Germany; 7 Hematology and Stem Cell Transplantation, Helios Clinic Berlin-Buch, Berlin, Germany; 8 Department of Hematology, Inselspital, Bern University Hospital, University of Bern, Bern, Switzerland; 9 Department of Hematology, Oncology, and Clinical Immunology, Heinrich-Heine University Düsseldorf, Düsseldorf, Germany; 10 Center for Integrated Oncology, Aachen Bonn Cologne Düsseldorf (CIO ABCD), Düsseldorf, Germany

**Keywords:** Myelodysplastic neoplasm, Iron overload, Genetic instability, γH2AX, Telomere, Serum ferritin

## Abstract

**Purpose:**

Serum ferritin is an independent prognostic marker in myelodysplastic neoplasms (MDS) and serves as a surrogate parameter for iron overload. Oxidative stress derived from iron overload may induce genomic damage, thereby promoting genetic instability and disease progression in MDS. We aimed to evaluate a possible association between iron overload via serum ferritin and various parameters for genetic instability in MDS.

**Methods:**

Fifty-one patients with confirmed MDS were analyzed and divided into three groups based on ferritin levels: non-elevated (≤ 275 µg/L), moderately elevated (> 275 and < 1000 µg/L), and highly elevated (≥ 1000 µg/L). Genetic instability was assessed by cytogenetic analysis and somatic mutation profiling. DNA double-strand breaks were quantified by γH2AX-foci in CD34+ peripheral blood cells, and telomere length was measured by flow-FISH.

**Results:**

Elevated serum ferritin was associated with increased cytogenetic abnormalities and somatic mutations at genomic regions commonly involved in MDS, higher levels of double-strand breaks, and shortened telomeres in granulocytes but not in lymphocytes. Markers of early-stage genetic instability, such as double-strand breaks and telomere shortening in granulocytes, were detectable at moderately elevated ferritin > 275 µg/L, whereas markers for advanced-stage genetic aberrations, including somatic mutations and cytogenetic aberrations, were more prominent at ferritin levels ≥ 1000 µg/L.

**Conclusion:**

These findings support the hypothesis that iron overload, reflected by elevated ferritin as surrogate parameter, may contribute to and/or increase genetic instability in MDS patients with ineffective hematopoiesis. Notably, correlations were observed at ferritin levels below current thresholds for initiating iron chelation therapy, indicating clinical relevance early in the disease course.

**Supplementary Information:**

The online version contains supplementary material available at 10.1007/s00432-026-06457-1.

## Introduction

Myelodysplastic neoplasms (MDS) are a heterogeneous group of clonal stem cell disorders with ineffective hematopoiesis, varying degrees of dysplasia and cytopenia, and an increased risk for leukemic transformation. The disease is driven by genetic instability, reflected by clonal bone marrow cells harboring typical cytogenetic and molecular aberrations associated with disease phenotype and prognosis (Schanz et al. 2012; Bernard et al. 2022), as well as the accumulation of these aberrations over time (Mazzeo et al. 2024).

With increasing disease duration, MDS patients often show increasing serum ferritin (SF) levels, mainly due to red blood cell (RBC) transfusions required for anemia, and partly due to ineffective erythropoiesis, which enhances iron absorption through suppressed hepcidin (Gattermann 2018). Higher SF is an adverse prognostic marker in transfusion-dependent MDS patients (Malcovati et al. 2006) and associated with relevant comorbidities. Besides elevated SF, further markers of iron overload (IOL) are non-transferrin-bound iron (NTBI) and the redox active forms of labile plasma iron (LPI). There is evidence that IOL promotes genetic instability via iron‑driven oxidative stress and reactive oxygen species (ROS), which cause oxidative DNA damage such as double-strand breaks, trigger error‑prone repair pathways, and thereby foster mutagenesis, chromosomal instability, clonal evolution, and progression to AML (Gattermann and Rachmilewitz 2011; Leitch and Gattermann 2019; Leitch 2025).

Double-strand breaks are increased in MDS and reflect genetic instability (Xiao et al. 2013). Moreover, critically shortened telomeres can result from increased cellular turnover and/or ROS mediated DNA damage. They may contribute to increasing genetic instability, particularly through chromosome rearrangements (Brümmendorf and Balabanov 2006). Telomeres are shortened to a variable degree in defined subtypes of MDS (Beier et al. 2015; Schneider et al. 2016). This phenomenon correlates with the complexity of the karyotype and severity of the disease (Hartmann et al. 2005; Vajen et al. 2013). The effects of IOL on genetic instability have been studied not only in MDS but particularly in hemochromatosis, where SF is elevated over prolonged periods and iron is accumulated preferably in the liver. Patients with hereditary hemochromatosis are at increased risk of developing hepatocellular carcinoma, associated with a high frequency of *TP53* mutations also in non-tumorous liver cells (Vautier et al. 1999; Hussain et al. 2000).

Several studies examined the relationship between IOL, ROS generation, and oxidative stress in MDS and report a positive correlation between SF levels and ROS (Ghoti et al. 2007; Pimkova et al. 2014; Chan et al. 2021). A mouse model demonstrated a direct relationship between IOL, increased ROS production and reduced clonogenic capacity of hematopoietic stem and progenitor cells (Chai et al. 2015). An impaired proliferation of erythroid progenitor cells was shown in MDS patients with elevated SF (Hartmann et al. 2013). However, most studies emphasize increased ROS and DNA damage markers as supporting evidence, while direct measurements of genetic instability in MDS patients with IOL remain rare. Recently, several murine studies have provided direct mechanistic insights. In MDS mouse models, IOL increases double‑strand breaks in hematopoietic stem and progenitor cells, indicated by elevated γH2AX. Iron restriction reduces this damage, underscoring a causal role of iron in genetic instability (Antypiuk et al. 2025). Likewise, mice with genetically induced IOL exhibit increased chromosomal instability and aneuploidy in bone marrow cells (Duarte et al. 2023).

Clonal MDS cells with intrinsic genetic instability may be more susceptible to additional genetic damage in the context of IOL, potentially mediated by oxidative stress. It is therefore possible that increased ROS could contribute to the accumulation of further genomic abnormalities in an already unstable preleukemic clone. To explore this potential relationship, we analyzed markers of IOL and genomic instability in patients with MDS.

## Material and methods

### Patients

A total of 51 patients with de novo MDS treated in four German centers (Göttingen, Leipzig, Hamburg, and Düsseldorf) between 2013 and 2015 were included. Active infections were excluded. All treatment regimens except for current iron chelation were allowed. Patient characteristics are shown in Table 1.

**Table 1:** Patient characteristics and comparison of patients with non-elevated, moderately elevated, and elevated serum ferritin levels

	All patients	Subgroups			
		Non-elevated serum ferritin (≤ 275 µg/L); group (1)	Moderately elevated serum ferritin (> 275 µg/L, < 1000 µg/L); group (2)	Highly elevated serum ferritin (≥ 1000 µg/L); group (3)	*p* value*
Number of patients	51	26	10	15	
Male/female	32/19	14/12	8/2	10/5	0.373
Age (years), median [range]	74 [26–85]	71 [26–85]	72 [61–83]	74 [50–79]	0.236
*WHO 2022, n (%)*					
MDS-5q**	4 (7.8%)	3 (11.5%)	0 (0.0%)	1 (6.7%)	0.111
MDS-SF3B1	3 (5.9%)	1 (3.8%)	1 (10.0%)	1 (6.7%)	
MDS-LB	21 (41.2%)	14 (53.8%)	4 (40.0%)	3 (20.0%)	
MDS-IB1	11 (21.6%)	4 (15.4%)	2 (20.0%)	5 (33.3%)	
MDS-IB2	7 (13.7%)	1 (3.8%)	2 (20.0%)	4 (26.7%)	
CMML-1	3 (5.9%)	3 (11.5%)	0 (0.0%)	0 (0.0%)	
CMML-2	1 (2.0%)	0 (0.0%)	1 (10.0%)	0 (0.0%)	
MDS/MPN-NOS	1 (2.0%)	0 (0.0%)	0 (0.0%)	1 (6.7%)	
IPSS-R karyotype category***, n (%)					
Very good	4 (7.8%)	4 (15.4%)	0 (0.0%)	0 (0.0%)	0.026
Good	31 (60.8%)	17 (65.4%)	7 (70.0%)	7 (46.7%)	
Intermediate	9 (17.6%)	5 (19.2%)	1 (10.0%)	3 (20.0%)	
Poor	7 (13.7%)	0 (0.0%)	2 (20.0%)	5 (33.3%)	
Very poor	0 (0.0%)	0 (0.0%)	0 (0.0%)	0 (0.0%)	
IPSS-M category****					
Very low	14 (27.5%)	11 (42.3%)	3 (30.0%)	0 (0.0%)	0.007
Low	17 (33.3%)	10 (38.5%)	3 (30.0%)	4 (26.7%)	
Moderate low	4 (7.8%)	2 (7.7%)	1 (10.0%)	1 (6.7%)	
Moderate high	2 (3.9%)	1 (3.8%)	0 (0.0%)	1 (6.7%)	
High	8 (15.7%)	2 (7.7%)	2 (20.0%)	4 (26.7%)	
Very high	6 (11.8%)	0 (0.0%)	1 (10.0%)	5 (33.3%)	
*Blood counts, median [range]*					
Hemoglobin (g/dl)	10.7 [7.0–14.9]	11.9 [7.6–14.9]	11.3 [8.6–14.0]	8.8 [7.0–11.2]	2.61 × 10^–5^
Platelets (/nl)	115 [4–1578]	136 [19–1578**]	106 [35–309]	60 [4–476]	0.635
ANC (/nl)	2.21 [0.10–12.65]	2.57 [0.22–12.65]	2.65 [0.58–5.84]	1.59 [0.10–9.35]	0.481
Bone marrow blasts (%)	3 [0–16]	2.5 [0–12]	4 [0–16]	7 [2–15]	0.014
Serum ferritin (µg/L)	256 [8–3872]	65 [8–256]	512 [283–899]	1714 [1005–3872]	8.61 × 10^–10^
*Transfusion dependence, median [range]*					
RBC per month	0.0 [0.0–10.0]	0.0 [0.0–1.0]	0.0 [0.0–0.7]	1.3 [0.0–10.0]	3.08 × 10^–7^
*Time from first diagnosis to analysis within the study (months)*					
	15 [0–177]	22.5 [3–77]	11 [0–79]	8 [0–177]	0.062

*Evaluated by the following tests: continuous variables by Kruskal–Wallis test, categorical variables (sex, WHO and IPSS-R karyotype category) by Fisher’s exact test (Monte Carlo simulation, B = 10,000);

Dunn’s test with Bonferroni-adjusted *p* values for pairwise comparisons; Hemoglobin (g/dl): group 1 vs 3, *p* = 2.56 × 10⁻^5^; 2 vs 3, *p* = 0.004; 1 vs 2, *p* = 1.000; Bone marrow blasts (%): see Table 2; serum ferritin (µg/L): group 1 vs 2: *p* = 0.003; group 1 vs 3: *p* = 7.45 × 10^-10^; group 2 vs 3: *p* = 0.118; RBC per month: group 1 vs 3, *p* = 2.61 × 10^⁻7^; group 2 vs 3, *p* = 6.55 × 10^⁻4^; group 1 vs 2, 1.000;

**One patient with isolated del(5q) and a previously described *JAK2* V617F mutation that was not detectable at the time of inclusion into the study

***According to Schanz et al. (2011)

****According to Bernard et al. (2022)

ANC, absolute neutrophile count; CMML, chronic myelomonocytic leukemia; IPSS-R, revised international prognostic scoring system for MDS; RBC, red blood concentrates; MDS, myelodysplastic neoplasm; MDS-5q, MDS with low blasts and isolated 5q deletion; MDS-IB, MDS with increased blasts; MDS-LB, MDS with low blasts; MDS/MPN-NOS, myelodysplastic/myeloproliferative neoplasm, not otherwise specified; MDS-SF3B1, MDS with low blasts and *SF3B1* mutation; WHO, world health organization

### Genetic alterations

Cytogenetic aberrations were analyzed by conventional chromosomal banding and by interphase fluorescence in situ hybridization (FISH) on immunomagnetically enriched CD34+ peripheral blood (PB) cells, using established protocols (Haase et al. 1995; Braulke et al. 2013). Molecular karyotyping was performed using the CytoScan HD Array (Affymetrix, Santa Clara, CA, USA) according to previously published methods (Ganster et al. 2015). Total genomic alteration size was calculated from array data, representing the cumulative size of all genomic gains, losses, and copy-number-neutral loss-of-heterozygosity events (Cluzeau et al. 2013). Number of cytogenetic aberrations per patient was derived from total number of distinct abnormalities identified by chromosomal banding, FISH, and array-based karyotyping. Identical aberrations detected by more than one method (e.g., del(5q) by both banding and FISH) were counted only once. Mutational profiling included Sanger sequencing of 16 recurrently mutated MDS-associated genes. Targeted deep sequencing of *TP53* was performed using the Nextera XT2 Sample Preparation Kit and sequencing on a MiSeq platform (Illumina, San Diego, CA, USA) as previously reported (Martin et al. 2018). Detailed genetic information for each patient is provided in Supplementary Table 1. The genes analyzed are shown in Supplementary Table 3. The IPSS-M risk categories in Table 1 are reported based on our gene panel, using the best-case scenario provided by the IPSS-M calculator (Bernard et al. 2022).

### γH2AX-foci

To detect double-strand breaks, immunomagnetically enriched CD34+ PB cells were stained using a primary γH2AX antibody (Phospho-Histone H2A.X (Ser139) (20E3) Rabbit mAB, Cell Signaling Technologies, Danvers, MA, USA) and a secondary fluorescent antibody (Anti-rabbit IgG Fab2 Alexa Fluor (R) 555 Molecular Probe, Cell Signaling Technologies, Danvers, MA, USA). CD34-positivity was confirmed using a primary CD34+ antibody (CD34+ FITC (8G12), Becton Dickinson, Franklin Lakes, NJ, USA) and a secondary fluorescent antibody (Alexa Fluor 488 goat anti-mouse IgH, Invitrogen, Waltham, MA USA). Counterstaining was performed using DAPI (4',6-diamidino-2-phenylindole) (Supplementary Fig. 1). In a fluorescent microscope ≥ 200 cells per patient were evaluated.

### Telomere length

Flow-FISH on PB samples processed within 48 h was carried out according to previously described protocols (Weidner et al. 2014; Beier et al. 2015; Werner et al. 2015; Ferreira et al. 2020; Tometten et al. 2023). Briefly, samples were analyzed in triplicate with and without Alexa488-(C3TA2) PNA (Panagene, South Korea). Cow thymocytes with a previously determined telomere length served as internal control to calculate telomere length in kilo base pairs (kb). Cow thymocytes, granulocytes and lymphocytes were identified based on forward scatter properties and LDS 751 fluorescence. Age-adjusted telomere length was given in comparison to healthy controls as delta telomere length in kb (Weidner et al. 2014; Beier et al. 2015; Werner et al. 2015; Ferreira et al. 2020; Tometten et al. 2023).

### Circulating nitrogen plasma pool

Circulating nitric oxide (NO) plasma pool was determined by measurement of nitrite and nitrate using the NOx analyzer ENO-20 (Eicom) and of nitroso species using reductive gas phase chemiluminescence detection (Rassaf et al. 2003).

### Transferrin bound iron and labile plasma iron

Total NTBI was assessed by FeROS eLPI, LPI, a fraction of NTBI, by FeROS LPI assay (CellTrend, Luckenwalde, Germany).

### Statistical analyses

Group comparisons of continuous variables were performed using the Wilcoxon test for two-group comparisons and the Kruskal–Wallis test for comparisons involving three groups. Dunn’s post hoc test was used for pairwise comparisons with Bonferroni adjustment where appropriate. Categorical variables were compared using Fisher’s exact test, with Monte-Carlo simulation in cases of low expected cell counts. Correlations between variables were assessed using Spearman’s rank correlation coefficient. Odds ratios (OR), 95% confidence intervals (CI), and *p* values were obtained from logistic regression models, adjusted for the influence of medullary blasts. Hazard ratios (HR) and 95% confidence intervals (CI) were estimated using Cox proportional hazards regression, with *p* values obtained from Wald tests. Where relevant, models were adjusted for bone marrow blast percentage. *P* values < 0.05 were considered statistically significant. Statistical analyses were performed using the software R version R 4.5.2 (2025).

## Results

### Clinical characteristics

The study cohort comprised 51 patients with different MDS subtypes (32 males, 19 females; median age 74 years, range 26–85). Normal SF level was defined as 22–275 µg/L. Based on the upper limit of normal and the established threshold of 1000 µg/L for initiation of iron chelation therapy, patients were stratified into three groups: non-elevated SF (≤ 275 µg/L; group 1; N = 26), moderately elevated SF (> 275 µg/L and < 1000 µg/L; group 2; N = 10), and highly elevated SF (≥ 1000 µg/L; group 3; N = 16).

Across these groups, higher SF was associated with lower hemoglobin (median 11.9, 11.3, and 8.8 g/dl in groups 1–3, respectively; *p* = 2.61 × 10^–5^), higher bone marrow blasts (median 2.5%, 4%, and 7%; *p* = 0.014), and more RBC transfusions in the three months prior to study inclusion (median 0.0, 0.0, and 1.3 units; *p* = 3.08 × 10^–7^). Moreover, increasing SF levels were associated with higher IPSS-R karyotype categories (*p* = 0.026) and higher IPSS-M categories (*p* = 0.007). In contrast, no statistically significant differences between SF groups were observed for sex, age, WHO subtype, platelet count, absolute neutrophil count (ANC), or time from first diagnosis to study inclusion (Table 1).

At or prior to study inclusion, three patients received immunomodulating therapy (two with non-elevated SF and one with highly elevated SF), with treatment initiation 25–36 months prior to inclusion. Six patients were treated with hypomethylating agents (HMA), including one with moderately elevated SF and five with highly elevated SF. HMA treatment was initiated 2–18 months prior to study inclusion. One patient with moderately elevated SF at inclusion had discontinued iron chelation therapy 4.8 years before study entry.

### Parameters associated with high SF

We investigated the association of SF with bone marrow blasts and with parameters of genetic instability, genetic alterations, and NO levels. Bone marrow blasts showed a strong correlation with SF (correlation coefficient = 0.563, *p* = 4.73 × 10^–5^). Among the parameters of genetic instability, the average number of γH2AX-foci in CD34+ PB cells showed a positive correlation with increasing SF (correlation coefficient = 0.494, *p* = 0.039), while age-adjusted telomere length in PB granulocytes showed a negative correlation (correlation coefficient = -0.585, *p* = 0.002). As expected and consistent with lineage-specific differentiation defects, no significant correlation was observed between age-adjusted telomere length in PB lymphocytes and SF. The number of genetic alterations also correlated with SF: number of cytogenetic aberrations (correlation coefficient = 0.395, *p* = 0.004), number of mutated MDS genes (correlation coefficient = 0.381, *p* = 0.007), total number of genetic alterations (molecular and cytogenetic; correlation coefficient = 0.503, *p* = 0.0003), and total genomic alteration size (correlation coefficient = 0.387, *p* = 0.007). In contrast, none of the NO parameters showed a significant correlation with SF (Table 2).

**Table 2:** Parameters associated with IOL-related parameters

	Correlation coefficient (*p* value*)	Non-elevated serum ferritin (≤ 275 µg/L); group (1)	Moderately elevated serum ferritin (> 275 µg/L, < 1000 µg/L); group (2)	Highly elevated serum ferritin (≥ 1000 µg/L) group (3)	*p* value**					
					global	(1) vs (2)	(1) vs (3)	(2) vs (3)	≤ 275 µg/L vs. > 275 µg/L	< 1000 µg/L vs. ≥ 1000 µg/L
Bone marrow blasts (%)	0.563 (4.73 × 10^–5^)	2.5 [0–12]; n = 22	4 [0–16]; n = 10	7 [2–15]; n = 14	0.014	0.169 (0.506)	0.004 (0.011)	0.258 (0.773)	0.007	0.010
*Genetic instability*										
Average number of γH2AX- foci in CD34+ PB cells	0.494 (0.039)	1.9 [0.5–6.8]; n = 8	6.1 [3.4–8.6]; n = 3	5.2 [2.1–10.8]; n = 7	0.054	0.057 (0.171)	0.038 (0.114)	0.756 (1.00)	0.016	0.151
Telomere length in PB granulocytes (kb)	−0.585 (0.002)	0.480 [−3.127; 5.318]; n = 13	−1.611 [−2.445; −0.869]; n = 3	−1.519 [−4.062; 1.311]; n = 10	0.026	0.060 (0.181)	0.016 (0.048)	0.771 (1.00)	0.006	0.053
Telomere length in PB lymphocytes (kb)	−0.217 (0.297)	0.387 [−2.540; 5.006]; n = 13	−1.233 [−1.293; −0.730]; n = 3	−0.553 [−4.110; 1.816]; n = 9	0.147	0.051 (0.153)	0.706 (1.00)	0.103 (0.309)	0.295	0.890
*Genetic aberrations*										
Number of cytogenetic aberrations	0.395 (0.004)	1 [0–3]; n = 26	0 [0–5]; n = 10	2 [0–3]; n = 15	0.044	0.613 (1.00)	0.013 (0.040)	0.132 (0.396)	0.048	0.015
Number of molecular mutated genes	0.381 (0.007)	0 [0–3]; n = 23	2 [0–3]; n = 10	2 [0–5]; n = 15	0.026	0.571 (1.00)	0.007 (0.022)	0.099 (0.298)	0.033	0.009
Total number of genetic alterations (molecular and cytogenetic)	0.503 (0.0003)	2 [0–5]; n = 23	2 [0–7]; n = 10	3 [1–7]; n = 15	0.005	0.378 (1.00)	0.001 (0.003)	0.068 (0.205)	0.008	0.002
Total genomic alteration size (Mb)	0.387 (0.007)	0 [0–155]; n = 23	0 [0–146]; n = 10	76 [0–248]; n = 15	0.014	0.896 (1.00)	0.007 (0.022)	0.021 (0.063)	0.105	0.009
*NO levels*										
Nitrite (nM)	0.256 (0.151)	102 [20–305]; n = 16	122 [105–395]; n = 4	100 [35–435]; n = 13	0.468	0.220 (0.660)	0.624 (1.00)	0.379 (1.090)	0.397	0.912
Nitrate (µM)	0.041 (0.822)	30 [14–107]; n = 16	27 [16–37]; n = 4	31 [13–72]; n = 13	0.786	0.495 (1.00)	0.935 (1.00)	0.539 (1.00)	0.759	0.912
Nitroso species (nM)	0.111 (0.558)	10 [5–20]; n = 15	5 [5–25]; n = 3	10 [5–40]; n = 12	0.781	0.944 (1.00)	0.513 (1.00)	0.645 (1.00)	0.611	0.499

*Evaluated using the Spearman correlation coefficient. No correction for multiple comparisons

**Evaluated by the Wilcoxon test for the comparison of two groups and the Kruskal–Wallis test for the comparison of three groups (global *p* value; Dunn’s test with non-adjusted *p* values and Bonferroni-adjusted *p* values in brackets). No correction for multiple comparisons besides the values in brackets

PB, peripheral blood; IOL, iron overload; kb, kilo base pairs; Mb, mega base pairs; n, number; nM, nano molar; µM, micro molar

**Fig. 1:**
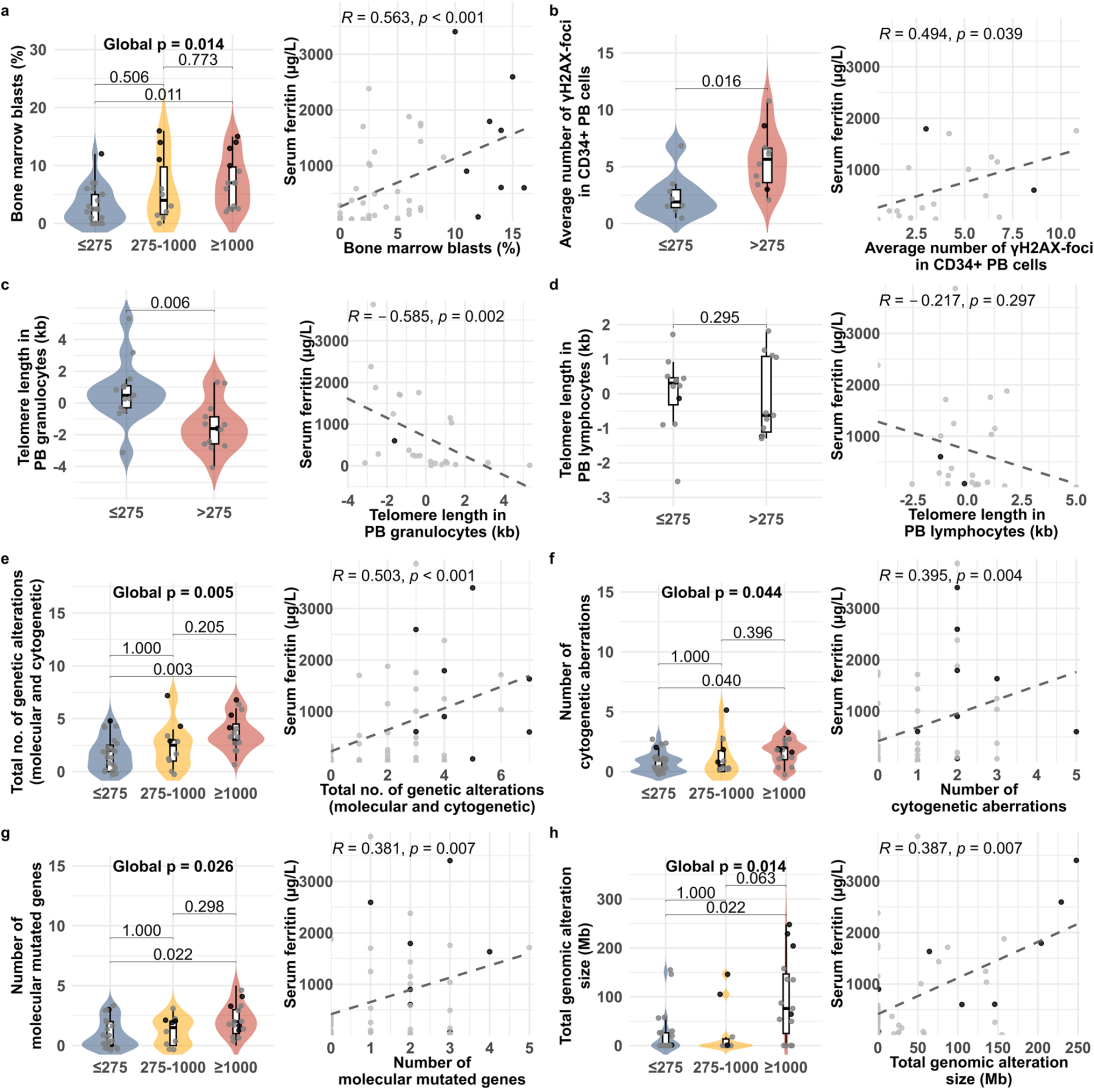
Parameters associated with high ferritin were assessed in patients with non-elevated (≤ 275 µg/L), moderately elevated (> 275 and < 1000 µg/L), and highly elevated SF (≥ 1000 µg/L). Shown are **a** bone marrow blasts; markers for early-stage genetic instability such as **b** average number of γH2AX-foci in CD34+ peripheral blood cells, **c** age-adjusted telomere length in peripheral blood granulocytes (kilo base pairs, kb), **d** age-adjusted telomere length in peripheral blood lymphocytes (kb); and markers for advanced-stage genetic aberrations such as **e** total number of genetic alterations (molecular and cytogenetic), **f** number of cytogenetic aberrations, **g** number of molecularly mutated genes, and **h** total genomic alteration size (mega base pairs, Mb). Black points indicate patients with bone marrow blasts ≥ 10% and grey points those with bone marrow blasts < 10%

We next compared these candidate parameters across the three previously defined SF groups. Parameters reflecting NO levels did not differ significantly between the three SF groups. In contrast, among the markers of genetic instability, the average number of γH2AX-foci in CD34+ PB cells increased with higher SF (group 1: 1.9 foci (range 0.5–6.8); group 2: 6.1 foci (3.4–8.6); group 3: 5.2 foci (range 2.1–10.8)). This trend yielded a global *p* value of 0.054. Pairwise comparisons revealed a significant difference between group 1 and group 3 (*p* = 0.038), as well as between group 1 and the combined groups 2 and 3 (*p* = 0.016). Age-adjusted telomere length in PB granulocytes decreased with increasing SF, with median values of 0.480 kb (range -3.127; 5.318) in group 1, -1.611 kb (range -2.445; -0.869) in group 2, and -1.519 kb (range -4.062; 1.311) in group 3. The global *p* value was 0.026, with a significant difference observed between group 1 and group 3 (*p* = 0.016), as well as between group 1 and the combined groups 2 and 3 (*p* = 0.006). In contrast, age-adjusted telomere length in PB lymphocytes did not differ significantly between the three groups. Finally, bone marrow blasts increased with SF (group 1: 2.5% (range 0–12); group 2: 4% (range 0–16); group 3: 7% (range 2–15), global *p* value 0.014) (Table 2, Fig. 1).

Regarding genetic aberrations, the number of all aberration types analyzed increased with rising SF. The number of cytogenetic aberrations showed a trend towards significance across groups, with a median of 1 aberration (range 0–3) in group 1, 0 aberrations (range 0–5) in group 2, and 2 aberrations (0–3) in group 3 (*p* = 0.044). The most notable differences were observed between group 1 and group 3 (*p* = 0.013) and when comparing the combined groups 1 and 2 to group 3 (*p* = 0.015). The number of molecularly mutated genes increased across groups (group 1: 0 aberration (range 0–3); group 2: 2 aberrations (range 0–3); group 3: 2 aberrations (range 0–5); global *p* = 0.026), with significant differences between group 1 and group 3 (*p* = 0.007), and between the combined groups 1 and 2 and group 3 (*p* = 0.009). The total number of genetic alterations (molecular and cytogenetic combined) also rose with higher SF (group 1: 2 aberrations (range 0–5); group 2: 2 aberrations (range 0–7); group 3: 3 aberrations (range 1–7); global *p* = 0.005), with significant differences between group 1 and group 3 (*p* = 0.001) and between groups with SF < 1000 µg/L and ≥ 1000 µg/L (*p* = 0.002). Similarly, the total genomic alteration size increased from 0 kb (range 0–155) in group 1, to 0 kb (range 0–146) in group 2, and 76 kb (range 0–248) in group 3 (global *p* = 0.014), with significant differences between group 1 and group 3 (*p* = 0.007) and between patients with SF < 1000 µg/L and ≥ 1000 µg/L (*p* = 0.009, Table 2, Fig. 1). No correction for multiple testing was applied for the values in Table 2, as all tests were considered individually, except for the comparison across the three groups, where Bonferroni-adjusted *p* values are indicated in round brackets.

As bone marrow blast counts were significantly correlated with SF, we additionally estimated odds ratios (OR) for all parameters using logistic regression, including bone marrow blast percentage as a covariate for adjustment (Table 3). When comparing patients with SF ≤ 275 µg/L to those with SF > 275 µg/L, the OR for the average number of γH2AX-foci in CD34+ PB cells was 9.87 (95% CI: 1.10–164.28, *p* = 0.063), and thus borderline significant after adjustment for bone marrow blast count. The OR for age-adjusted telomere length in PB granulocytes was 0.06 (95% CI: 0.00–0.44, *p* = 0.012) after adjustment. In contrast, the comparison between patients with SF < 1000 µg/L and those with SF ≥ 1000 µg/L did not yield statistically significant ORs for γH2AX-foci and age adjusted telomere length in granulocytes. Moreover, the age-adjusted telomere length in PB lymphocytes was not significantly associated with SF level in either comparison (SF ≤ 275 µg/L vs. > 275 µg/L, or SF < 1000 µg/L vs. ≥ 1000 µg/L, Table 3).

**Table 3:** Logistic regression models for serum ferritin ≤ 275 vs. > 275 and for serum ferritin < 1000 vs. ≥ 1000

	Serum ferritin ≤ 275 µg/L vs. > 275 µg/L						Serum ferritin < 1000 µg/L vs. ≥ 1000 µg/L					
	Unadjusted model			Adjusted for blast counts			Unadjusted model			Adjusted for blast counts		
	OR	95% CI	*p* value	OR	95% CI	*p* value	OR	95% CI	*p* value	OR	95% CI	*p* value
*Genetic instability*												
Average number of γH2AX- foci in CD34+ PB cells	7.00	0.97–72.56	0.069	9.87	1.10–164.28	0.063	4.38	0.62–42.44	0.158	4.36	0.60–43.82	0.164
Telomere length in PB granulocytes (kb)	0.09	0.01–0.50	0.010	0.06	0.00–0.44	0.012	0.26	0.04–1.31	0.115	0.27	0.04–1.39	0.130
Telomere length in PB lymphocytes (kb)	0.22	0.04–1.12	0.080	1.16	0.91–1.63	0.085	0.62	0.11–3.22	0.572	0.65	0.12–3.44	0.607
*Genetic aberrations*												
Number of cytogenetic aberrations	1.86	1.09–3.50	0.034	1.62	0.89–3.11	0.125	1.77	1.04–3.25	0.047	1.53	0.83–2.91	0.179
Number of molecular mutated genes	1.71	1.06–2.94	0.037	1.46	0.88–2.56	0.160	2.06	1.23–3.81	0.011	1.88	1.08–3.55	0.034
Total number of genetic alterations (molecular and cytogenetic)	1.66	1.16–2.55	0.010	1.50	1.01–2.35	0.056	1.80	1.23–2.90	0.006	1.70	1.12–2.80	0.020
Total genomic alteration size (Mb)	1.95	0.62–6.31	0.255	1.26	0.36–4.43	0.714	3.73	1.04–15.80	0.053	2.75	0.69–12.33	0.162
*NO levels*												
Nitrite (nM)	1.00	1.00–1.01	0.314	1.00	1.00–1.01	0.220	1.00	0.99–1.01	0.733	1.00	0.99–1.01	0.545
Nitrate (µM)	0.98	0.95–1.01	0.276	0.98	0.95–1.01	0.282	0.99	0.96–1.02	0.609	0.99	0.96–1.02	0.660
Nitroso species (nM)	1.06	0.95–1.21	0.349	1.09	0.95–1.31	0.303	1.04	0.94–1.17	0.436	1.04	0.93–1.21	0.532

Reference group is non-elevated SF (≤ 275 µg/L) and non-elevated/moderately elevated SF (< 1000 µg/L), respectively. No correction for multiple comparisons

PB, peripheral blood; CI = confidence interval; kb, kilo base pairs; Mb, mega base pairs; nM, nano molar; OR, odds ratio; µM, micro molar

The ORs for all parameters of genetic aberrations were significantly increased when comparing SF < 1000 µg/L vs. ≥ 1000 µg/L. After adjustment for bone marrow blast count, the OR for the number of molecularly mutated genes was 1.88 (95% CI 1.08–3.55, *p* = 0.034); for the total number of genetic alterations (molecular and cytogenetic combined) the OR was 1.70 (95% CI 1.12–2.80, *p* = 0.020); and for the total genomic alteration size the OR was 2.75 (95% CI 0.69–12.33, *p* = 0.162). No significant associations were observed for any of the NO level parameters in either comparison (Table 3). Separate analyses for patients with SF ≤ 275 µg/L and ≥ 1000 µg/L were not conducted due to insufficient subgroup sizes.

Finally, increased bone marrow blast counts were significantly associated with shorter survival. Hazard ratio (HR) for patients with bone marrow blasts ≥ 10% compared to < 10% was 5.01 (95% CI 1.86–13.53, *p* = 0.001). Elevated SF was also significantly associated with reduced overall survival. The HR for SF > 275 µg/L vs. ≤ 275 µg/L was 5.83 (95% CI 2.06–16.45, *p* = 0.001), and for SF ≥ 1000 µg/L vs. ≤ 275 µg/L the HR was 3.97 (95% CI 1.51–10.44, *p* = 0.005). The association between elevated SF and shorter survival remained statistically significant even after adjustment for bone marrow blast count (Supplementary Table 2).

Besides bone marrow blast count and SF, one additional parameter showed a borderline significant association with overall survival. Analyses were conducted using groups defined by median splits of the respective parameters to enable comparison of two approximately equal-sized groups. When age-adjusted telomere length in PB granulocytes was dichotomized at -0.43 kb (low: < -0.43 kb vs. high: ≥ -0.43 kb), the resulting HR was 4.12 (95% CI 0.96–17.59, *p* = 0.056). However, after adjustment for bone marrow blasts and SF, age-adjusted telomere length in PB granulocytes did not remain significantly associated with survival (Supplementary Table 2). No significant association with survival was observed for any of the remaining parameters.

## Discussion

The most pronounced differences in early markers of genomic instability, including double-strand breaks and telomere shortening, were observed between patients with SF above and below 275 µg/L. In contrast, advanced genetic aberrations, such as the number of somatic mutations, cytogenetic abnormalities, and total genomic alteration burden, became evident only when comparing patients with SF ≥ 1000 µg/L vs. < 1000 µg/L. This indicates that while early indicators of genomic instability may be affected already at moderately elevated iron levels, the accumulation of detectable genetic alterations likely requires a higher degree and/or longer duration of iron exposure. The extended time interval since patient recruitment allowed to estimate overall survival. This is particularly important in MDS, where disease progression and iron-related complications often evolve over several years. To reflect the biological and clinical heterogeneity of MDS, we included patients across different disease stages and treatment regimes. However, treatment heterogeneity and survival were not primary endpoints of this study, and besides age-adjusted telomere length in PB granulocytes, we did not observe a statistically significant association between the analyzed genetic parameters on overall survival. This may be attributable to limited statistical power regarding survival analysis, the small proportion of patients with very high-risk and thus prognostically very poor cytogenetic profiles (Supplementary Table 1), and the fact that iron chelation and other treatments initiated after study inclusion were not systematically assessed. Registry-based studies have demonstrated a survival benefit associated with iron chelation therapy (Hoeks et al. 2020), which was subsequently confirmed in a prospective randomized trial (Angelucci et al. 2020). The adverse impact of IOL induced genetic instability on survival may become apparent only with sustained exposure over the course of the disease.

A limitation of our study is the assessment of IOL primarily by SF as surrogate marker for IOL, although patients with active infections were excluded and measurements therefore are reliable. Transferrin saturation, LPI, and eLPI were only available for a subset of patients. In line with previous studies, LPI was detectable in some transfusion-dependent patients (Swart et al. 2017; Hoeks et al. 2021), but in our patient group the number of measurable values was insufficient for robust statistical analysis.

As our cohort did not include patients with severe IOL and thus lacked very high SF or sufficiently elevated LPI/eLPI levels for robust analysis, intracellular analysis of oxidative stress may have been a more sensitive marker for this patient group. However, due to limited sample material, we did not measure direct biomarkers of oxidative stress, such as 8-hydroxy-2′-deoxyguanosine (8-OHdG), a specific oxidative DNA damage product. Instead, we analyzed metabolites of NO, given that oxidative stress can reduce NO bioavailability. Although plasma nitrite and nitrate are not considered direct indicators of oxidative stress, previous studies reported reduced nitrite levels in MDS patients compared with healthy controls (Pimkova et al. 2014) and described an association between nitrite and SF in MDS (Souza et al. 2013). In contrast, we did not observe an association between NO metabolites and SF, likely due to the limited number of patients with available NO measurements.

The primary aim of our study was to assess associations between SF levels and genetic markers. Although our findings suggest possible causal relationships, establishing causality would require longitudinal and/or prospective functional studies. Many of the analyzed variables are biologically and clinically interrelated, and our univariate statistical approach cannot fully account for complex confounding structures, even though adjustment for blast counts was included. Due to the limited sample size, robust multivariable modeling including multiple potential confounders was not feasible.

Although we lacked patients with very high SF, we detected markers of genetic instability like γH2AX-foci and shortened telomers in our cohort. Telomere shortening is a marker of cellular aging, replicative stress, and oxidative damage and may promote genetic instability and malignant progression. In our cohort, age-adjusted telomere shortening in PB granulocytes was significantly associated with increased SF, supporting a role of iron-driven, ROS-mediated damage to telomeres. Telomere shortening affected the myeloid but not the lymphoid compartment, consistent with previous reports indicating that telomere attrition in MDS is largely confined to the malignant myeloid clone (Beier et al. 2015). Along this line, accelerated telomere shortening might contribute to disease progression in MDS.

Recently, a preclinical study provided causal evidence in a MDS mouse model that genetically induced iron excess increases baseline double-strand breaks, measured by γH2AX, in the stem/progenitor compartment, and pharmacologic or genetic iron restriction reduces γH2AX levels (Antypiuk et al. 2025). Accordingly, we could show that SF correlates with the number of γH2AX-foci in CD34+ PB progenitor cells of MDS patients. Although γH2AX-foci are a sensitive indicator of DNA damage, they are not a fully specific marker of double-strand breaks (Löbrich et al. 2010). We hypothesize that these γH2AX-foci contribute to the acquirement of genetic aberrations in MDS. An association between γH2AX-foci and genetic aberrations in malignant myeloid diseases was shown before when the number of γH2AX-foci was increased in AML with complex karyotypes or unfavorable gene mutations, but not in MDS (Popp et al. 2017). Recently, a genetically induced IOL mouse model developed MDS-like features, providing evidence for a causal link between IOL and MDS, including increased chromosomal instability as assessed by karyotyping (Duarte et al. 2023).

An increased number of chromosomal aberrations, MDS-associated gene mutations and total genomic alteration size was also observed in our study in MDS patients with elevated SF, suggesting that iron promotes the occurrence of these alterations. The mutational burden observed in our cohort may be underestimated, as our analysis was restricted to 16 genes assessed by Sanger sequencing and *TP53* by NGS. In line with our results and the recently described mouse model, an association of SF with 8-OHdG levels and chromosomal aberrations in MDS patients was described (Kikuchi et al. 2012). Although we detected several genetic aberrations, molecular *TP53* mutations were infrequent, observed in only 2 of 52 patients. This is consistent with previous reports showing that *TP53* mutations are mainly restricted to advanced subtypes with complex karyotypes or del(5q) (Bernard et al. 2022). As evidence for IOL-induced *TP53* mutations largely derives from hepatocytes of patients with hereditary hemochromatosis (Vautier et al. 1999; Hussain et al. 2000), exhibiting markedly higher SF levels than observed in our cohort, *TP53* mutations may represent a late event requiring prolonged and more severe IOL.

Previous studies indicate that IOL not only promotes genetic instability but also impairs hematopoiesis in MDS. Suppression of erythroid colony forming capacity illustrated the inhibitory effects of IOL on immature hematopoietic cells (Hartmann et al. 2013). Consistently, reduced clonogenic capacity of hematopoietic stem and progenitor cells was observed in a non-MDS mouse model in which IOL was induced by iron dextran (Chai et al. 2015). Moreover, genetically induced IOL mouse models develop MDS-like features, including dysplasia (Duarte et al. 2023).

In summary, our data support the clinical notion that IOL contributes to MDS progression through a stepwise process. Iron-driven oxidative stress may induce double-strand breaks and accelerate telomere shortening already at moderately elevated iron levels. With disease progression and sustained or more severe iron exposure, this genomic stress may facilitate the acquisition of cytogenetic and molecular aberrations and thus genetic progression. In parallel, IOL impairs normal hematopoiesis, further promoting clonal selection and expansion of genetically altered cells. The combination of impaired hematopoiesis, ongoing genomic instability, and accumulation of genetic lesions may ultimately contribute to disease progression and leukemic transformation in MDS. These observations provide a biological framework linking IOL to clonal evolution and underscore the potential relevance of timely iron chelation strategies in the management of MDS.

## Supplementary Information

Below is the link to the electronic supplementary material.

Supplementary Material 1

## Data Availability

The data that support the findings of this study are available from the corresponding author upon reasonable request.
