# Supplementary material for: High serum ferritin is associated with genetic instability in myelodysplastic neoplasms
Source: J Cancer Res Clin Oncol. 2026 Apr 9;152(4):85. doi: 10.1007/s00432-026-06457-1 (PMC13066071; doi:10.1007/s00432-026-06457-1)
Supplement: Supplementary file 1 — Supplementary Material 1 [file 432_2026_6457_MOESM1_ESM.docx]

**Supplementary Information Legends**

**Supplementary Table 1** Cytogenetic and molecular alterations

**Supplementary Table 2** Association of IOL-related parameters with overall survival

**Supplementary Table 3 Genes included in the Sanger sequencing panel**

**Supplementary Fig. 1** γH2AX-foci (red, Phospho-Histone H2A.X (Ser139) (20E3) Rabbit mAB) indicating double-strand breaks in immunomagnetically enriched CD34+ peripheral blood cells (green, CD34+ FITC (8G12)) counterstained with DAPI (4',6-diamidino-2-phenylindole, blue)

**Supplementary Information**

**Supplementary Table 1** Cytogenetic and molecular alterations

| Patient ID | Cytogenetic aberrations | Number of cytogenetic aberrations | Molecular mutated genes | Number of molecular mutated genes | Total number of mutations (molecular and cytogenetic) | Serum ferritin [µg/L] |
| --- | --- | --- | --- | --- | --- | --- |
| 17 | N | 0 | none | 0 | 0 | 8 |
| 6 | -X | 1 | none | 0 | 1 | 12 |
| 16 | N | 0 | none | 0 | 0 | 19 |
| 5 | N | 0 | none | 0 | 0 | 24 |
| 7 | del(13q) | 1 | none | 0 | 1 | 30 |
| 14 | N | 0 | *SRSF2*, *TET2* | 2 | 2 | 30 |
| 47 | hmz(7q) | 1 | *ASXL1*, *RUNX1*, *SRSF2* | 3 | 4 | 32 |
| 39 | N | 0 | *SF3B1*, *TET2*, *ZRSR2* | 3 | 3 | 38 |
| 38 | del(5q) | 1 | none | 0* | 1 | 42 |
| 50 | del(5q) | 1 | *TP53* normal | NA | NA | 42 |
| 45 | N | 0 | *SRSF2*, *TET2* | 2 | 2 | 55 |
| 21 | -Y | 1 | *TET2* | 1 | 2 | 60 |
| 36 | -Y | 1 | none | 0 | 1 | 61 |
| 20 | N | 0 | *TP53* normal | NA | NA | 69 |
| 4 | N | 0 | none | 0 | 0 | 72 |
| 13 | hmz(6q), hmz(22q), ‑Y | 3 | *ZRSR2* | 1 | 4 | 76 |
| 8 | +8, del(21q) | 2 | *ASXL1*, SR*S*F2, *TET2* | 3 | 5 | 78 |
| 10 | N | 0 | none | 0 | 0 | 102 |
| 15 | -Y, +14 | 2 | *TP53* (VAF 5%) | 1 | 3 | 104 |
| 41 | N | 0 | *ASXL1*, *TET2* | 2 | 2 | 124 |
| 43 | del(12p) | 1 | *ASXL1*, *RUNX1*, *SRSF2* | 3 | 4 | 146 |
| 35 | N | 0 | none | 0 | 0 | 197 |
| 34 | del(3p), -Y | 2 | none | 0 | 2 | 198 |
| 23 | del(5q) | 1 | not available | NA | NA | 236 |
| 18 | N | 0 | none | 0 | 0 | 242 |
| 25 | +8 | 1 | *SRSF2* | 1 | 2 | 256 |
| 26 | N | 0 | none | 0 | 0 | 283 |
| 48 | N | 0 | none | 0 | 0 | 328 |
| 49 | hmz(11q) | 1 | none | 0 | 1 | 371 |
| 12 | +1, der(1;7), +21 | 3 | none | 0 | 3 | 375 |
| 29 | N | 0 | *ASXL1*, *SRSF2*, *TET2* | 3 | 3 | 492 |
| 42 | N | 0 | *SF3B1* | 1 | 1 | 533 |
| 46 | N | 0 | *SF3B1*, *TET2* | 2 | 2 | 586 |
| 19 | del(20q), +12, i(12q), hmz(11q), hmz(21q) | 5 | *RUNX1*, *SRSF2* | 2 | 7 | 601 |
| 37 | +8 | 1 | *DNMT3A*, *U2AF1* | 2 | 3 | 606 |
| 11 | dup(21q), del(Xp) | 2 | *DNMT3A*, *TET2* | 2 | 4 | 899 |
| 24 | hmz(7q) | 1 | *ASXL1*, *EZH2* | 2 | 3 | 1005 |
| 31 | t(1;13), dup(1q), hmz(22q) | 3 | *ASXL1*, *SF3B1*, *TET2* | 3 | 6 | 1036 |
| 33 |  | 0 | *ASXL1*, *EZH2* | 2 | 2 | 1150 |
| 28 | hmz(3q), del(5q), del(17q) | 3 | *TP53* (VAF 16% and 54%) | 1 | 4 | 1246 |
| 44 | hmz(2p) | 1 | *TET2*, *U2AF1* | 2 | 3 | 1434 |
| 9 | t(2;6), del(6p), del(7q) | 3 | *DNMT3A*, *RUNX1*, *SRSF2*, *TET2* | 4 | 7 | 1633 |
| 27 | N | 0 | *SRSF2* | 1 | 1 | 1701 |
| 3 | hmz(7q) | 1 | *ASXL1*, *EZH2*, *RUNX1*, *SF3B1*, *TET2* | 5 | 6 | 1714 |
| 32 | N | 0 | *ASXL1*, *TET2*, *U2AF1* | 3 | 3 | 1757 |
| 30 | hmz(7p), +8 | 2 | *ASXL1*, *U2AF1* | 2 | 4 | 1793 |
| 1 | -7, del(20q) | 2 | none | 0 | 2 | 1879 |
| 51 | -7 | 2 | *ASXL1*, *U2AF1* | 2 | 4 | 2380 |
| 2 | -7, hmz(11q) | 2 | *CBL* | 1 | 3 | 2593 |
| 40 | hmz(2p), -7 | 2 | *DNMT3A*, *SRSF2*, *IDH2* | 3 | 5 | 3404 |
| 22 | del(9q), del(11q) | 2 | *ASXL1* | 1 | 3 | 3872 |

hmz, homozygous (copy number neutral loss of heterozygosity); NA, not available (if sequencing was not performed or if only *TP53* was specifically analyzed); N, normal karyotype; VAF, variant allele frequency;

* the previously described *JAK2* V617F mutation was not detectable at the time of inclusion into the study

Supplementary Table 2 Association of IOL-related parameters with overall survival

|  | **Univariable model** | | | |  | | **Adjusted model (blasts)** | | | |
| --- | --- | --- | --- | --- | --- | --- | --- | --- | --- | --- |
|  | **HR** | **95% CI** | ***p* value** |  | | **HR** | | **95% CI** | ***p* value** |  |
| Bone marrow blasts (≥10% vs. <10%) | 5.01 | 1.86-13.53 | 0.001 |  | | - | | - | - |  |
| Serum ferritin   >275 µg/L vs. ≤275 µg/L  ≥1000 µg/L vs. ≤275 µg/L | 5.83 3.97 | 2.06-16.45 1.51-10.44 | 0.001 0.005 |  | | 4.17 3.50 | | 1.34-12.98 1.30-9.43 | 0.014 0.014 |  |
| **Genetic instability** |  |  |  |  | |  | |  |  |  |
| Average number of γH2AX-foci in CD34+ PB cells  high (≥3.5 foci) vs. low (<3.5 foci) | 1.74 | 0.49-6.22 | 0.394 |  | | - | | - | - |  |
| Telomere length in PB granulocytes (kb)  low (< -0.43 kb) vs. high (≥ -0.43 kb) | 4.12 | 0.96-17.59 | 0.056 |  | | 3.74 | | 0.84-16.65 | 0.084* |  |
| Telomere length in PB lymphocytes (kb)  low (<0.22 kb) vs. high (≥0.22 kb) | 1.37 | 0.29-6.52 | 0.689 |  | | - | | - | - |  |
| **Genetic aberrations** |  |  |  |  | |  | |  |  |  |
| Number of cytogenetic aberrations   (≥1 aberration vs. no aberration) | 1.14 | 0.52-2.53 | 0.740 |  | | - | | - | - |  |
| Number of molecular mutated genes  (≥1 aberration vs. no aberration) | 2.36 | 0.93-6.00 | 0.072 |  | | - | | - | - |  |
| Total number of genetic alterations (molecular and   cytogenetic, ≥3 alterations vs. 0-2 alterations) | 2.13 | 0.94-4.82 | 0.069 |  | | - | | - | - |  |
| Genomic alterations by molecular karyotyping   (abnormal vs. normal) | 1.27 | 0.56-2.89 | 0.574 |  | | - | | - | - |  |

Cox regression analyses for parameters of genetic instability and genetic aberrations were conducted using groups defined by median splits of the respective parameters to allow comparison of two approximately equal-sized groups. Adjusted models were fitted only for parameters that were statistically significant or showed a borderline significance in the univariable analysis. Adjustment for blasts: ≥10% vs. <10% bone marrow blasts; adjustment for serum ferritin (only for age adjusted telomere length in granulocytes): ≤275 µg/L, >275 µg/L and <1000 µg/L, ≥1000 µg/L.

* Model adjusted for blasts and for serum ferritin: HR 2.92, 95% CI 0.58-14.76, *p* value 0.195

**Supplementary Table 3** Genes included in the Sanger sequencing panel

| *ASXL1* |
| --- |
| *CBL* |
| *DNMT3* |
| *ETV6* |
| *EZH2* |
| *IDH1* |
| *IDH2* |
| *JAK2* |
| *KRAS* |
| *NRAS* |
| *RUNX1* |
| *SF3B1* |
| *SRSF2* |
| *TET2* |
| *TP53* |
| *U2AF1* |
| *ZRSR2* |


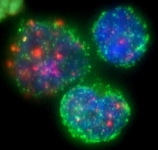


**Supplementary Fig. 1** γH2AX-foci (red, Phospho-Histone H2A.X (Ser139) (20E3) Rabbit mAB) indicating double-strand breaks in immunomagnetically enriched CD34+ peripheral blood cells (green, CD34+ FITC (8G12)) counterstained with DAPI (4',6-diamidino-2-phenylindole, blue)
